# Physiologically Based Pharmacokinetic Modeling to Assess Antiretroviral–BTK Inhibitor Interactions and Provide Recommendations for Co-Administration Regimens

**DOI:** 10.3390/pharmaceutics18040465

**Published:** 2026-04-10

**Authors:** Lu Chen, Xiaoxiao Wang, Lixian Li, Yi Yang, Yao Liu, Wanyi Chen

**Affiliations:** 1 Department of Pharmacy, Chongqing University Cancer Hospital, Chongqing 400030, China; luchen.23@cqu.edu.cn (L.C.); xiaoxiao.wang@cqu.edu.cn (X.W.); lilixian@cqu.edu.cn (L.L.); 20220701085g@stu.cqu.edu.cn (Y.Y.); 2 State Key Laboratory of Neurology and Oncology Drug Development, Nanjing 210023, China; 3 Chongqing Key Laboratory of Translational Research for Cancer Metastasis and Individualized Treatment, Department of Hematology-Oncology, Chongqing University Cancer Hospital, Chongqing 400030, China; 4 School of Medical, Chongqing University, Chongqing 400044, China

**Keywords:** HIV-related lymphoma (HRL), antiretroviral drugs, BTK inhibitors, drug–drug interactions, physiologically based pharmacokinetic (PBPK)

## Abstract

**Objectives**: The co-administration of Bruton’s tyrosine kinase (BTK) inhibitors with antiretroviral drugs is challenging due to potential drug–drug interactions (DDIs). However, clinical trials specifically assessing such DDIs are lacking. We aimed to evaluate DDIs between the BTK inhibitors ibrutinib, zanubrutinib and acalabrutinib with representative antiretroviral drugs and to provide dose adjustment strategies using physiologically based pharmacokinetic (PBPK) models. **Methods**: PBPK models were developed in PK-Sim software. Model performance was verified by comparing simulated pharmacokinetic parameters and DDI magnitudes with probe drugs (midazolam or maraviroc) with reported clinical data. The validated models were subsequently applied to assess DDIs and explore dose adjustment strategies. **Results**: The developed PBPK model accurately describes the pharmacokinetics of each drug. Darunavir/ritonavir substantially increased the maximum plasma concentration (C_max_) of ibrutinib, zanubrutinib, and acalabrutinib by 496%, 312%, and 160%, respectively. In contrast, efavirenz reduced C_max_ by 43%, 33%, and 37%, respectively, while etravirine caused smaller decreases of 5%, 0%, and 10%. Based on these predictions, recommended dose adjustment strategies include ibrutinib 105 mg once daily, zanubrutinib 40 mg twice daily, and acalabrutinib 50 mg twice daily when co-administered with darunavir/ritonavir or ibrutinib 980 mg once daily, zanubrutinib 240 mg twice daily, and acalabrutinib 150 mg twice daily when co-administered with efavirenz. No dose adjustment is required with etravirine. **Conclusions**: The PBPK models accurately predicted the in vivo pharmacokinetics of ibrutinib, zanubrutinib, acalabrutinib, and those of the antiretrovirals darunavir/ritonavir, efavirenz, and etravirine, and the DDIs between them. The dose adjustment strategies provided information valuable to the optimization of antineoplastic therapy in HIV-related lymphoma (HRL) patients.

## 1. Introduction

Globally, human immunodeficiency virus (HIV) remains a major public health concern. The number of patients with HIV worldwide continues to grow, with approximately 37.7 million patients diagnosed in 2020 and 39 million patients in 2022 [1]. In addition, nearly 680,000 people died from diseases associated with acquired immunodeficiency syndrome [2]. Individuals with HIV are living longer due to the widespread availability of combination antiretroviral therapy (cART).

People with HIV are more likely to develop various diseases, including malignancies. Severe immunosuppression in advanced HIV infection is a risk factor for the occurrence of a variety of malignancies, which have become the leading cause of death in persons with HIV [3,4]. Lymphomas represent one of the most common malignancies among persons with HIV [5]. Since 2017, the incidence of HIV-related lymphoma (HRL) has exceeded that of Kaposi’s sarcoma [6].

The combination of cART and antineoplastic therapy has markedly prolonged survival rates in individuals with HIV-associated malignancies [7]. Nevertheless, lymphoma survival rates still differ between patients with HIV and the general population, which emphasizes the need for wider dissemination of cART protocols [5].

Bruton’s tyrosine kinase (BTK) inhibitors, including ibrutinib, zanubrutinib, and acalabrutinib, have demonstrated promising antitumor activity in the treatment of lymphoma [8]. In the comprehensive treatment of HRL patients, combinations of BTK inhibitors and cART have emerged as means of antitumor treatment and control HIV disease progression. However, antiretroviral drugs are mostly potent inhibitors or inducers of cytochrome P450 enzymes, and patients taking cART regimens are at a high risk of drug–drug interactions (DDIs) with antineoplastics [9,10], which may increase therapeutic toxicity or decrease efficacy [3,11]. Increased drug toxicity may lead to the interruption of antineoplastic or antiretroviral drug administration or a reduction in drug doses, increasing the risk of viral rebound and tumor recurrence [11]. For example, Corona et al. [12] reported that lopinavir/ritonavir reduced the clearance of irinotecan (CPT11) by 47% by inhibiting the metabolic enzyme CPT11, resulting in a dramatic increase in the area under the plasma concentration–time curve (AUC) of CPT11 metabolites by 200%. Denise et al. [13] reported that two patients who received paclitaxel during antiretroviral therapy developed life-threatening toxicities due to CYP3A enzyme-mediated DDI.

Currently, the clinical data on DDIs between BTK inhibitors and antiretroviral drugs remain limited. Many clinical trials exclude people with HIV due to ethical considerations, so there is little possibility of running formal clinical trials investigating the interactions between cART and BTK inhibitors. Therefore, there is an urgent need to find an alternative to clinical trials to evaluate potential DDIs and provide evidence for BTK inhibitor dose adjustment in HIV patients.

Physiologically based pharmacokinetic (PBPK) modeling is a modelling approach that can be used to predict DDIs in the absence of clinical data [14]. This approach integrates in vitro data (e.g., physicochemical property parameters) with systematic data that describes the population of interest (e.g., anatomical and physiological characteristics and demographics) to simulate the pharmacokinetics of drugs [15]. This strategy has been increasingly accepted by regulatory agencies as an alternative for evaluating potential DDIs between a substrate drug and an interacting drug [16].

Therefore, this study aimed to predict the magnitude of DDIs between BTK inhibitors (ibrutinib, zanubrutinib, and acalabrutinib) and antiretroviral drugs (darunavir/ritonavir, efavirenz, and etravirine) through PBPK modeling. In addition, PBPK models were applied to simulate ibrutinib, zanubrutinib, and acalabrutinib dose adjustments to achieve therapeutic drug exposure when these agents were co-administered with antiretrovirals.

## 2. Materials and Methods

PBPK models were performed using the Open Systems Pharmacology software suite (version 12), including PK-Sim and MoBi. Each model was constructed and optimized to match the observed pharmacokinetic parameters (AUC and maximum plasma concentration (C_max_)) of the target drug. Plasma concentration–time data were digitized from published literature using the GetData Graph Digitizer (version 2.25). All clinical data used for model development and verification were obtained from published studies.

### 2.1. PBPK Model Development and Verification for BTK Inhibitors

The development of PBPK models for BTK inhibitors (ibrutinib, zanubrutinib, and acalabrutinib) was based on clinical pharmacokinetic parameters, physicochemical property parameters, and data from in vitro experiments [17,18,19]. The basic physicochemical property parameters entered into the models included molecular weight, solubility, the acid dissociation constant (pKa), the octanol–water partition coefficient (logP), fraction unbound in gut (fu,gut), fraction unbound in plasma (f_up_), effective permeability (P_eff_), and the blood-to-plasma concentration ratio (R_bp_). In vitro data describing the physicochemical characteristics of BTK inhibitors are summarized in Table 1. Similarly, data describing the metabolism and elimination of the drugs by different enzyme isoforms were obtained from the literature, and intrinsic clearance values for these metabolic processes were incorporated into the model, as shown in Table 1.

Based on the published studies [17,18,19], for oral absorption, the advanced dissolution, absorption, and metabolism (ADAM) model was applied in ibrutinib and zanubrutinib, while a first-order absorption model was utilized in acalabrutinib. Distribution was simulated using a full PBPK model for ibrutinib and zanubrutinib, and a minimal PBPK model for acalabrutinib. After the PBPK models were performed, simulations were performed at doses of 420 mg of ibrutinib, 80 mg of zanubrutinib, and 100 mg of acalabrutinib, based on the routine regimens of these drugs in the clinic. Model verification was performed by comparing simulated pharmacokinetic profiles with observed data from published clinical studies [20,21,22].

The prediction accuracy of simulated pharmacokinetic parameters AUC and C_max_ was quantitatively evaluated by fold-error values. When the observed value > predicted value, fold error = observed/predicted; otherwise, fold error = predicted/observed. The performance of the PBPK model was considered acceptable when fold error values fell within a two-fold range, which is consistent with commonly used PBPK model evaluation criteria.

### 2.2. PBPK Model Development and Verification for Antiretroviral Drugs

The PBPK models developed for antiretroviral drugs were similar to those of the BTK inhibitors. Ritonavir, darunavir, efavirenz, and etravirine act as inhibitors or inducers of cytochrome P450 enzymes. The corresponding physicochemical property data input into the models are summarized in Table 2. For the oral absorption process, the ADAM model was applied to darunavir and etravirine, whereas a first-order absorption model was used for ritonavir and efavirenz. Distribution was simulated using a full PBPK model for darunavir and etravirine, and a minimal PBPK model for ritonavir and efavirenz. The oral absorption and distribution models were derived from the published studies [23,24,25,26,27,28,29]. The data describing the metabolism and elimination of the drugs through different enzyme isoforms were available in the published studies [23,24,25,26,27,28,29] and are listed in Table 2. After the PBPK models were constructed, simulations were performed at doses of 200 mg of ritonavir, 600 mg of darunavir, 600 mg of efavirenz, and 200 mg of etravirine based on the routine regimens of these drugs in the clinic. To verify the accuracy of the PBPK models, simulated pharmacokinetic parameters and plasma concentration profiles were compared with clinical data [30,31,32,33], and prediction performance was evaluated using fold error values.

### 2.3. DDI Simulation Design

First, steady-state plasma concentrations were simulated in a virtual population receiving the following regimens for 14 days: ibrutinib 560 mg once daily, zanubrutinib 160 mg twice daily, acalabrutinib 100 mg twice daily, darunavir/ritonavir 800/100 mg once daily, efavirenz 600 mg once daily, or etravirine 200 mg twice daily. Second, to validate the DDI model, the inhibitory and inductive potential of darunavir/ritonavir, efavirenz, and etravirine was assessed using CYP3A4 probe substrates (midazolam and maraviroc), and simulated interactions were compared with data from published DDI studies [34,35,36]. Finally, the validated model was used to simulate potential DDIs between BTK inhibitors (ibrutinib, zanubrutinib, and acalabrutinib) and antiretroviral drugs. Additionally, optimal dose adjustments for BTK inhibitors were explored to achieve comparable exposures to those observed in the absence of antiretroviral drugs.

In the darunavir/ritonavir regimen, ritonavir was assumed to be the dominant CYP3A4 inhibitor, whereas darunavir was assumed to provide only limited additional inhibition under the same conditions. This simplification was adopted to avoid potential overestimation of overlapping CYP3A4 inhibitory effects.

## 3. Results

### 3.1. Model Development and Validation of BTK Inhibitors and Antiretrovirals

**Figure 1:**
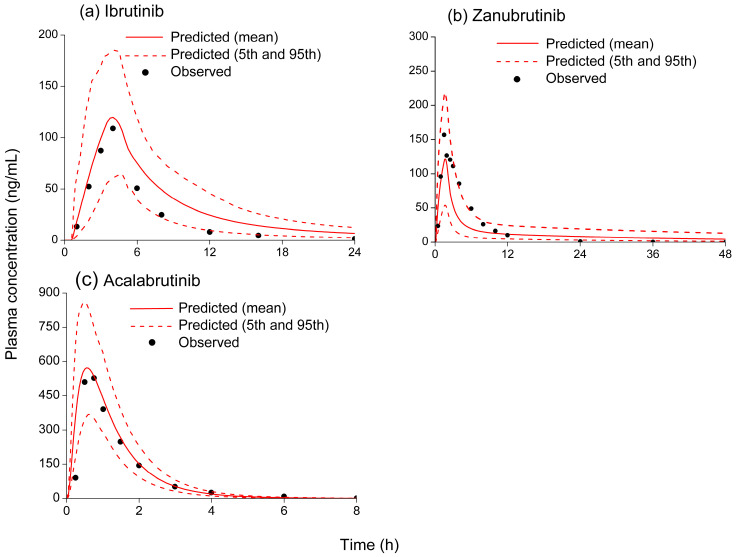
Observed (symbols) and simulated (solid red lines) plasma concentration–time profiles of ibrutinib, zanubrutinib, and acalabrutinib: (**a**) 420 mg of ibrutinib orally; (**b**) 80 mg of zanubrutinib orally; and (**c**) 100 mg of acalabrutinib orally. The dashed lines represent the 95th and 5th percentiles of the simulated concentrations.

**Figure 2:**
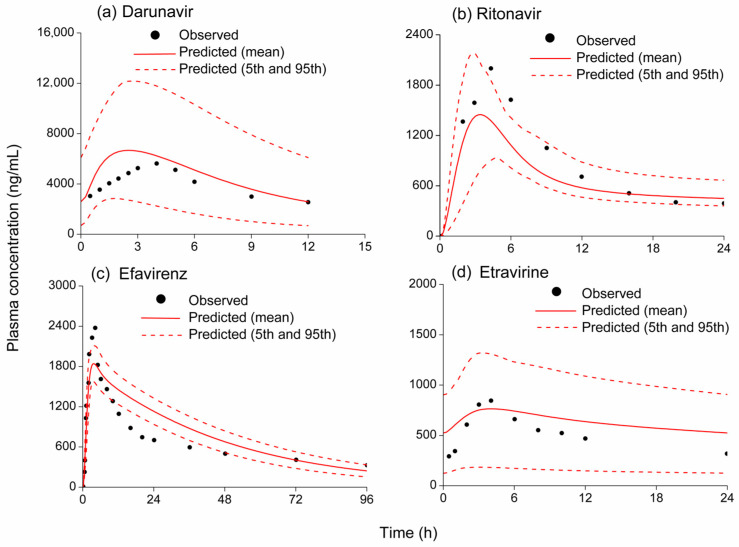
Observed (symbols) and simulated (solid red lines) plasma concentration–time profiles of darunavir, ritonavir, efavirenz, and etravirine: (**a**) 600 mg of darunavir orally; (**b**) 200 mg of ritonavir orally; (**c**) 600 mg of efavirenz orally; and (**d**) 200mg of etravirine orally. The dashed lines represent the 95th and 5th percentiles of the simulated concentrations.

The predictive accuracy of the PBPK models was assessed by comparing simulated plasma concentration–time profiles and pharmacokinetic data with corresponding observed profiles and data from clinical studies [20,21,22,30,31,32,33]. The simulated and observed plasma concentration–time curves of BTK inhibitors and antiretrovirals are shown in Figure 1 and Figure 2. It can be seen that the simulated pharmacokinetic profiles of BTK inhibitors and antiretrovirals predicted by the PBPK models were consistent with the observed profiles. The pharmacokinetic parameters (C_max_ and AUC) of ibrutinib, zanubrutinib, acalabrutinib, darunavir, ritonavir, efavirenz, and etravirine, and the fold error values are shown in Table 3. The C_max_ and AUC of each drug were comparable to the clinical data, indicating that the predictive performance of the established PBPK models is credible.

### 3.2. DDIs with Reference Probes

Since ritonavir is a more potent CYP3A4 inhibitor than darunavir, the inhibition of CYP3A4 by darunavir/ritonavir was described according to a competitive antagonistic model in which the additional CYP3A4 inhibition caused by darunavir is minimal when ritonavir is present.

As shown in Table 4, the simulated DDIs between darunavir/ritonavir, efavirenz, or etravirine with the probe substrates midazolam and maraviroc were generally comparable to published clinical data and fell within an acceptable two-fold range, indicating that the DDI models were adequate for exploratory prediction. However, the residual discrepancies between the simulated and observed interaction magnitudes may reflect uncertainty in perpetrator parameterization and the simplified representation of combined CYP3A4 inhibition in the darunavir/ritonavir regimen.

The simulated interaction between ritonavir and midazolam (100 mg of ritonavir plus 2 mg of midazolam) led to an increase in the midazolam area under the curve (AUC_0–inf_) by 20.57–fold, compared with the 15.02–fold increase reported in a clinical study [31]. In contrast, the simulated interaction between efavirenz (600 mg once daily) and maraviroc (100 mg twice daily) resulted in a 48% decrease in the maraviroc AUC_0–12_, compared with the 52% decrease reported in the clinical study by Abel et al. [35]. Finally, Kakuda et al. [36] evaluated the interaction between etravirine and maraviroc and reported a 53% decrease in maraviroc AUC_0–12_ when maraviroc was co-administered with etravirine (300 mg twice daily of maraviroc plus 200 mg twice daily of etravirine), while the corresponding simulation resulted in a 67% decrease.

### 3.3. DDIs with BTK Inhibitors

**Figure 3:**
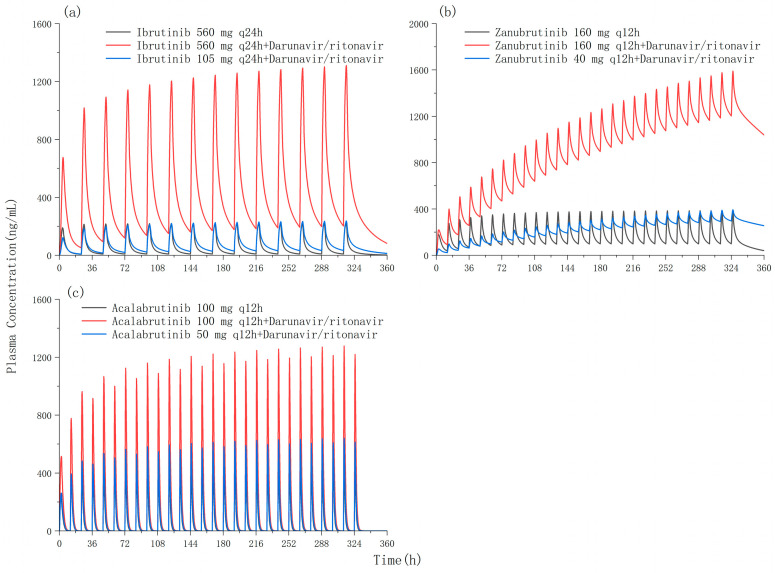
Simulated plasma concentration–time profiles of multiple doses (14 day doses) of (**a**) ibrutinib, (**b**) zanubrutinib, and (**c**) acalabrutinib in the absence and presence of darunavir/ritonavir (800/100 mg once daily).

The verified PBPK models were used to predict the magnitude of DDIs between BTK inhibitors (ibrutinib, zanubrutinib, and acalabrutinib) and antiretrovirals (darunavir/ritonavir, efavirenz, and etravirine). Darunavir/ritonavir had a major effect on the pharmacokinetics of ibrutinib, increasing AUC_0–τ_ and C_max_ 8.25- and 4.96-fold, respectively. At the standard regimen (560 mg once daily), the simulated AUC_0–τ_ and C_max_ of ibrutinib were equal to 1287.53 ng/mL·h and 219.96 ng/mL, respectively, and the DDIs with darunavir/ritonavir caused an increase to 11,905.99 ng/mL·h and 1310.47 ng/mL, respectively. A reduction in the ibrutinib dose was simulated, and a dose of 105 mg once daily was predicted to overcome the effect of darunavir/ritonavir on ibrutinib exposure (Table 5 and Figure 3a).

Similarly, darunavir/ritonavir increased the AUC_0–τ_ and C_max_ of zanubrutinib 6.38- and 3.12-fold, respectively. At the standard regimen (160 mg twice daily), the simulated AUC_0–τ_ and C_max_ of zanubrutinib were equal to 2144.00 ng/mL·h and 386.23 ng/mL, respectively, and the DDIs with darunavir/ritonavir caused an increase to 15,822.16 ng/mL·h and 1590.29 ng/mL. The simulated dosage reduced to 40 mg twice daily provided pharmacokinetic parameters comparable to those acquired with standard dosing without darunavir/ritonavir (Table 5 and Figure 3b).

In addition, darunavir/ritonavir increased the AUC_0–τ_ and C_max_ of acalabrutinib 1.90- and 1.60-fold, respectively. According to the simulated results, at the standard regimen (100 mg twice daily), the simulated AUC_0–τ_ and C_max_ of acalabrutinib were equal to 760.89 ng/mL·h and 468.62 ng/mL, respectively, and the DDIs with darunavir/ritonavir caused an increase to 2209.30 ng/mL·h and 1218.68 ng/mL. A dose adjustment to 50 mg twice daily was predicted to overcome the increase in darunavir/ritonavir on ibrutinib exposure (Table 5 and Figure 3c).

In contrast to darunavir/ritonavir, efavirenz and etravirine induced the metabolism of ibrutinib, zanubrutinib, and acalabrutinib, causing a decrease in drug exposure. Such a decrease was particularly marked in the case of efavirenz. In the case of efavirenz, a dose adjustment to 980 mg once daily of ibrutinib, 240 mg twice daily of zanubrutinib, and 150 mg twice daily of acalabrutinib was sufficient to provide pharmacokinetic parameters comparable to those obtained with standard dosing without efavirenz (Table 5 and Figure S1). In the case of etravirine, there is no need to adjust the dose of BTK inhibitors (Table 5 and Figure S2). As all simulated concentration–time curves declined to near zero in a similar pattern, we present the full profiles in Supplementary Figures S1 and S2 for clarity. The main outcomes of these changes in exposure are summarized in Table 5.

## 4. Discussion

The combination of cART and antineoplastic treatment has become relatively common in HRL patients, resulting in changes to drug exposure potentially affecting treatment outcomes. Thus, it is extremely important to clarify the magnitude of DDIs between BTK inhibitors and antiretrovirals. However, direct clinical data on DDIs between BTK inhibitors and antiretrovirals remain limited. Model-informed drug development (MIDD) is a powerful quantitative method and plays an indispensable role in drug development and regulatory review. The key application of MIDD is to provide information for clinical trial design, including dose selection and optimization. To date, PBPK modeling has mainly been used to evaluate DDIs and corresponding dose adjustments in regulatory submissions [37]. Therefore, this study aims to predict the DDIs between BTK inhibitors and antiretrovirals in order to inform rational dosing strategies for ibrutinib, zanubrutinib, or acalabrutinib when co-administered with darunavir/ritonavir, efavirenz, or etravirine in the treatment of HRL.

The developed PBPK models accurately describe the pharmacokinetics of BTK inhibitors (ibrutinib, zanubrutinib, and acalabrutinib) and antiretroviral drugs (darunavir/ritonavir, efavirenz, and etravirine) and their DDIs. Ibrutinib, zanubrutinib, and acalabrutinib are mainly metabolized by CYP3A4 and, to a lesser extent, by other isoenzymes [38]. Ritonavir and darunavir are also primarily metabolized by the CYP3A isoenzyme family [39]. Similarly, CYP2B6 and CYP3A4 are the main isozymes responsible for efavirenz metabolism, while etravirine is metabolized by CYP3A4 and CYP2C19 [39]. Additionally, ritonavir is a strong inhibitor and a weak inducer of CYP3A4, while darunavir is a weak inhibitor and inducer of CYP3A4 [39]. When considering drug interactions with darunavir/ritonavir, ritonavir was modeled as the primary perpetrator of CYP3A4 inhibition, whereas darunavir was assumed to contribute limited additional inhibition in the presence of ritonavir. This assumption was based on the well-established pharmacokinetic enhancement of ritonavir and its significantly stronger CYP3A4 inhibitory effect compared to darunavir [39].

According to the prescribing information, co-administration of ibrutinib or acalabrutinib with strong CYP3A4 inhibitors should be avoided, and a dose reduction is recommended when zanubrutinib needs to be co-administered with strong CYP3A4 inhibitors. Such recommendations are based on data that show a 24-fold increase in the AUC of ibrutinib when it was co-administered with ketoconazole, a 278% increase in the AUC of zanubrutinib when it was co-administered with itraconazole, and a 5.1-fold increase in the AUC of acalabrutinib when it was co-administered with itraconazole [40,41,42]. In our study, compared with taking alone, the C_max_ of ibrutinib, zanubrutinib, and acalabrutinib increased by 496%, 312%, and 160%, respectively, and the AUC increased by 825%, 638%, and 190%, respectively, when combined with darunavir/ritonavir. Based on our simulations, when co-administered with darunavir/ritonavir, the recommended dose reduction strategies for ibrutinib (105 mg once daily), zanubrutinib (40mg twice daily), and acalabrutinib (50 mg twice daily) provided comparable exposures to the standard regimen of ibrutinib, zanubrutinib, and acalabrutinib when used alone.

At drug concentrations observed in clinical practice, efavirenz exhibits a more pronounced induction of CYP3A4 than etravirine [43,44,45], as reflected by the magnitude of their interactions. Our data show that the exposure of ibrutinib, zanubrutinib, and acalabrutinib is reduced when co-administered with efavirenz. Compared with taking alone, the AUC of ibrutinib, zanubrutinib, and acalabrutinib decreased by 43%, 49%, and 37%, respectively, when combined with efavirenz. In this case, dose adjustments to ibrutinib (980 mg once daily), zanubrutinib (240mg twice daily), and acalabrutinib (150 mg twice daily) could achieve therapeutic concentrations in the presence of efavirenz. Etravirine is a weaker inducer of CYP3A4 and has a lower impact on the pharmacokinetics of ibrutinib, zanubrutinib, and acalabrutinib. Compared with taking alone, the AUC of ibrutinib, zanubrutinib, and acalabrutinib decreased by 9%, 1%, and 10%, respectively, when combined with etravirine. Therefore, no dose adjustment is needed when these agents are co-administered with etravirine.

From a clinical perspective, the dose adjustment strategies proposed in this study should be regarded as mode-informed recommendations rather than definitive dosing regimens for all HRL patients. Our study aims to provide rational recommendations for co-administration, support individualized clinical decision-making, and inform the design of future prospective DDI studies. In actual clinical practice, treatment decisions should also consider toxicity monitoring, treatment response, and the patient’s overall comorbidity and concomitant medication profile.

Our study retains several limitations. First, zanubrutinib and acalabrutinib are known substrates of transporters such as P-glycoprotein/ABCB1 and BCRP [46,47], and these transporters may also be modulated by antiretroviral drugs [48,49]. However, these processes were not incorporated into the present model because of insufficient quantitative data to support reliable transporter parameterization.

Second, the PBPK model in PK-Sim captured direct CYP3A4-mediated interactions but did not automatically incorporate indirect effects, such as changes in biochemical pathways or blood biochemical parameters, which would require additional data. In this study, we focused on CYP3A4-mediated interactions, which represent the primary changes in BTK inhibitor exposure.

In addition, simulations were conducted in healthy populations, whose inflammatory status, nutritional condition, and CYP3A4 activity may differ [50] and thus affect DDI magnitude in clinical practice.

Finally, uncertainty also arises from literature-derived model inputs, including the contribution of CYP3A4 to victim drug clearance. In addition, the model assumed that ritonavir was the dominant CYP3A4 inhibitor, with darunavir contributing limited additional inhibition. Since no formal sensitivity analysis was performed, the proposed regimens should be interpreted cautiously and, where possible, be accompanied by close clinical monitoring, toxicity assessment, and further clinical validation. Future work should evaluate the impact of parameters such as CYP3A4 expression and the inhibitory contribution of darunavir to further strengthen confidence in the proposed dose adjustment strategies.

## 5. Conclusions

In summary, we developed a PBPK model that predicted the DDIs between BTK inhibitors and antiretrovirals and provided dosing guidance for co-administration. Darunavir/ritonavir was predicted to substantially increase the AUC of ibrutinib, zanubrutinib, and acalabrutinib by 825%, 638%, and 190%, respectively. In contrast, efavirenz reduced the AUC by 43%, 49%, and 37%, respectively, while etravirine caused only a minimal effect. Based on these findings, recommended dose adjustment strategies include ibrutinib 105 mg once daily, zanubrutinib 40 mg twice daily, and acalabrutinib 50 mg twice daily when co-administered with darunavir/ritonavir, or ibrutinib 980 mg once daily, zanubrutinib 240 mg twice daily, and acalabrutinib 150 mg twice daily when co-administered with efavirenz. No dose adjustment is required when co-administered with etravirine. The model presented here provides a useful model-informed framework for evaluating potential DDIs and supporting dose selection in HRL. However, these predictions should be interpreted cautiously and require future clinical studies.

## Figures and Tables

**Table 1:** Physicochemical properties and pharmacokinetic data used for physiologically based pharmacokinetic (PBPK) modeling of Bruton’s tyrosine kinase (BTK) inhibitors [17,18,19].

Parameter	Ibrutinib [17]	Zanubrutinib [18]	Acalabrutinib [19]
pKa	3.78	3.3	3.54, 5.77
Molecular weight (g/mol)	440.5	471.55	465.5
R_bp_	0.827	0.804	0.787
P_eff_ (×10^−4^ cm/s)	-	0.9	5.4
logP	3.97	4.2	2.03
P_app,caco-2_ (×10^−6^ cm/s)	32.6	-	-
f_up_ (%)	2.7	5.82	2.6
HLM CL_int_ CYP3A4 (μL/min/mg)	8312	120	9.63 μL/min/pmol
Additional clearance HLM (μL/min/mg)	364.4	60	289.5
CYP3A4 V_max_ (pmol/min/pmol)	-	-	4.13
CYP3A4 K_m_ (μM)	-	-	2.78
CYP3A4 CL_int_ (μL/min/pmol)	-	-	8.14
CL_R_ (L/h)	0.004	0.5	1.33
P-gp concentration (μM)	-	-	0.16
P-gpV_max_ (μM/min)	-	-	50.0

pKa: acid dissociation constant; R_bp_: blood-to-plasma concentration ratio; P_eff_: effective permeability; logP: octanol–water partition coefficient; P_app,caco-2_: apparent permeability of Caco-2 cell line; f_up_: fraction unbound in plasma; HLM: human liver microsome; K_m_: Michaelis–Menten constant; V_max_: maximum rate of metabolism formation; CL_int_: intrinsic clearance; CL_R_: renal clearance.

**Table 2:** Physicochemical properties and pharmacokinetic data used for PBPK modeling of antiretroviral drugs [23,24,25,26,27,28,29].

Parameter	Ritonavir [23,24]	Darunavir [23,25]	Efavirenz [26,27]	Etravirine [28,29]
pKa	2	Neutral (2.4 (basic) 13.6 (acidic))	10.2	3.8
Molecular weight (g/mol)	721	547.7	315.7	453.28
R_bp_	0.58	0.64	0.74	0.7
logP	3.9	1.8	3.89 ^a^	5.2
P_app,caco-2_ (×10^−6^ cm/s)	2.1	5.5	2.5	6.5
f_up_ (%)	2	6	2	0.1 ^a^
CYP3A4 V_max_ (pmol/min/mg)	1.37	-	-	0.072, 0.067, 5.57, 0.166 (For CYP3A4-M1, CYP3A4-M2, CYP2C19 and CYP3A4-M3, respectively) (pmol/mg/min)
CYP3A4 K_m_ (µM)	0.07	-	-	5.83, 72.85, 7.33, 27.8
CYP3A5 V_max_ (pmol/min/mg)	1	-	-	-
CYP3A5 K_m_ (µM)	0.05	-	-	-
CYP2D6 V_max_ (pmol/min/mg)	0.7	-	-	-
CYP2D6 K_m_ (µM)	1	-	-	-
HLM CL_int_ CYP3A4 (μL/min/mg)	-	182	-	-
CYP1A2 CL_int_ (μL/min/pmol)	-	-	0.03	-
CYP2A6 CL_int_ (μL/min/pmol)	-	-	0.08	-
CYP3A4 CL_int_ (μL/min/pmol)	-		0.007	-
CYP2B6 CL_int_ (μL/min/pmol)	-	-	0.55	-
Additional clearance HLM (μL/min/mg)	75	-	-	900, 400 (PE Tool for 100 mg, 200 mg)
CL_R_ (L/h)	0.27	0.3	-	0.0006
CYP3A4/3A5 K_i_ (µM)		0.4	20.6	-
CYP3A4/3A5 K_app_ (µM)	0.25	-	-	-
CYP3A4/3A5 K_inact_ (1/h)	19.8	-	-	-
CYP3A4 Ind_max_	68.5	2.2	9.9	2.5
CYP3A4 Ind_50_	1	0.18 μM	3.8 μM	5.2 μM
CYP2B6 Ind_max_	-	-	6.2	-
CYP2B6 IndC_50_	-	-	1.2 μM	-

^a^ From DrugBank (https://go.drugbank.com/drugs/DB06414 (accessed on 14 August 2025)). pKa: acid dissociation constant; R_bp_: blood-to-plasma concentration ratio; logP: octanol–water partition coefficient; P_app,caco-2_: apparent permeability of Caco-2 cell line; f_up_: fraction unbound in plasma; K_m_: Michaelis–Menten constant; V_max_: maximum rate of metabolism formation; HLM: human liver microsome; CL_int_: intrinsic clearance; CL_R_: renal clearance; K_i_: concentration of inhibitor associated with half maximal inhibition; K_app_: concentration of mechanism-based inhibitor associated with half maximal inactivation rate; K_inact_: inactivation rate of the enzyme; Ind_max_: maximum fold of induction; IndC_50_: inducer concentration that support half maximal induction.

**Table 3:** Observed and predicted pharmacokinetic parameters of BTK inhibitors and antiretroviral drugs.

		C_max_ (ng/mL)	T_max_ (h)	AUC ^a^ (ng·h/mL)
Ibrutinib 420 mg	Observed	109	4	535
	Predicted	119.93	4	853.61
	Fold error	1.10	1.00	1.59
Zanubrutinib 80 mg	Observed	162.8	1.5	663.0
	Predicted	121.84	1.65	678.81
	Fold error	1.11	1.13	1.38
Acalabrutinib 100 mg	Observed	547	0.75	709.3
	Predicted	589.63	0.55	844.73
	Fold error	1.08	1.36	1.19
Ritonavir 200 mg	Observed	2000	4.3	18,700
	Predicted	1448.74	3.5	16,786.52
	Fold error	1.38	1.23	1.11
Darunavir 600 mg	Observed	5874	4	46,720
	Predicted	6672.64	2.5	50,343.23
	Fold error	1.14	1.6	1.08
Efavirenz 600 mg	Observed	2592	3	102,617
	Predicted	1841.30	3.75	87,186.58
	Fold error	1.41	1.25	1.18
Etravirine 400 mg	Observed	863	4	11,064
	Predicted	764.57	4	15,228.03
	Fold error	1.13	1.00	1.38

AUC: area under the plasma concentration–time curve; C_max_: maximum plasma concentration; T_max_: time-to-maximum plasma concentration. ^a^ AUC_0–24_ for ibrutinib, ritonavir, and etravirine. AUC_0–t_ for zanubrutinib. AUC_0–inf_ for acalabrutinib and efavirenz. AUC_0–12_ for darunavir.

**Table 4:** The predicted and observed geometric mean ratio of CYP3A4 probe drugs’ pharmacokinetic parameters in the absence and presence of antiretroviral drugs.

	C_max_ (ng/mL)			AUC (ng·h/mL)		
	Predicted Ratio	Observed Ratio	Fold-Error	Predicted Ratio	Observed Ratio	Fold-Error
Midazolam DDI with ritonavir	4.90	3.88	1.26	21.57	16.02 (AUC_inf_)	1.35
Maraviroc DDI with efavirenz	0.52	0.44	1.18	0.52	0.48 (AUC_0–12_)	1.08
Maraviroc DDI with etravirine	0.45	0.4	1.12	0.33	0.47 (AUC_0–12_)	1.42

DDI: drug–drug interaction; AUC: area under the plasma concentration–time curve; C_max_: maximum plasma concentration.

**Table 5:** Effect of darunavir/ritonavir, efavirenz, and etravirine on pharmacokinetic parameters of BTK inhibitors. Results are expressed as geometric mean ratio compared with standard dosing in absence of antiretrovirals.

	AUC_0–τ_	C_max_
Darunavir/Ritonavir 800/100 mg q24 h		
Ibrutinib 560 mg q24 h	9.25	5.96
Ibrutinib 105 mg q24 h	1.63	1.08
Zanubrutinib 160 mg q12 h	7.38	4.12
Zanubrutinib 40 mg q12 h	1.82	1.02
Acalabrutinib 100 mg q12 h	2.90	2.60
Acalabrutinib 50 mg q12 h	1.46	1.31
Efavirenz 600 mg q24 h		
Ibrutinib 560 mg q24 h	0.57	0.57
Ibrutinib 980 mg q24 h	1.00	0.87
Zanubrutinib 160 mg q12 h	0.51	0.67
Zanubrutinib 240 mg q12 h	0.95	1.10
Acalabrutinib 100 mg q12 h	0.63	0.63
Acalabrutinib 150 mg q12 h	0.78	0.87
Etravirine 200 mg q12 h		
Ibrutinib 560 mg q24 h	0.91	0.95
Zanubrutinib 160 mg q12 h	0.99	1.00
Acalabrutinib 100 mg q12 h	0.90	0.90

AUC: area under the plasma concentration–time curve; C_max_: maximum plasma concentration.

## Data Availability

No new data were created.

## Supplementary Materials

The following supporting information can be downloaded at: https://www.mdpi.com/article/10.3390/pharmaceutics18040465/s1, Figure S1: Simulated plasma concentration–time profiles of multiple doses (14 days doses) of (a) ibrutinib, (b) zanubrutinib, and (c) acalabrutinib in the absence and presence of efavirenz (600 mg once daily); Figure S2: Simulated plasma concentration–time profiles of multiple doses (14 days doses) of (a) ibrutinib, (b) zanubrutinib, and (c) acalabrutinib in the absence and presence of etravirine (200 mg twice daily).

## Abbreviations

The following abbreviations are used in this manuscript:

BTK	Bruton’s tyrosine kinase
DDIs	Drug–drug interactions
PBPK	Physiologically based pharmacokinetic
C_max_	Maximum plasma concentration
HIV	Human immunodeficiency virus
HRL	HIV-related lymphomas
cART	Combination antiretroviral therapy
AUC	Area under the plasma concentration–time curve
ADAM	Advanced dissolution, absorption, and metabolism
MIDD	Model-informed drug development

## References

[1] Swinkels H.M., Nguyen A.D., Gulick P.G. (2024). HIV and AIDS. StatPearls [Internet].

[2] Berhan A., Bayleyegn B., Getaneh Z. (2022). HIV/AIDS Associated Lymphoma: Review. Blood Lymphat. Cancer.

[3] Welz T., Wyen C., Hensel M. (2017). Drug Interactions in the Treatment of Malignancy in HIV-Infected Patients. Oncol. Res. Treat..

[4] Landgren O., Goedert J.J., Rabkin C.S., Wilson W.H., Dunleavy K., Kyle R.A., Katzmann J.A., Rajkumar S.V., Engels E.A. (2010). Circulating serum free light chains as predictive markers of AIDS-related lymphoma. J. Clin. Oncol..

[5] Carbone A., Vaccher E., Gloghini A. (2022). Hematologic cancers in individuals infected by HIV. Blood.

[6] Yarchoan R., Uldrick T.S. (2018). HIV-Associated Cancers and Related Diseases. N. Engl. J. Med..

[7] Gopal S., Patel M.R., Yanik E.L., Cole S.R., Achenbach C.J., Napravnik S., Burkholder G.A., Reid E.G., Rodriguez B., Deeks S.G. (2013). Temporal trends in presentation and survival for HIV-associated lymphoma in the antiretroviral therapy era. J. Natl. Cancer Inst..

[8] Tam C., Thompson P.A. (2024). BTK inhibitors in CLL: Second-generation drugs and beyond. Blood Adv..

[9] Hakkola J., Hukkanen J., Turpeinen M., Pelkonen O. (2020). Inhibition and induction of CYP enzymes in humans: An update. Arch. Toxicol..

[10] Stader F., Kinvig H., Battegay M., Khoo S., Owen A., Siccardi M., Marzolini C. (2018). Analysis of Clinical Drug-Drug Interaction Data To Predict Magnitudes of Uncharacterized Interactions between Antiretroviral Drugs and Comedications. Antimicrob. Agents Chemother..

[11] Cingolani A., Torti L., Pinnetti C., de Gaetano Donati K., Murri R., Tacconelli E., Larocca L.M., Teofili L. (2010). Detrimental clinical interaction between ritonavir-boosted protease inhibitors and vinblastine in HIV-infected patients with Hodgkin’s lymphoma. Aids.

[12] Corona G., Vaccher E., Sandron S., Sartor I., Tirelli U., Innocenti F., Toffoli G. (2008). Lopinavir-ritonavir dramatically affects the pharmacokinetics of irinotecan in HIV patients with Kaposi’s sarcoma. Clin. Pharmacol. Ther..

[13] Bundow D., Aboulafia D.M. (2004). Potential drug interaction with paclitaxel and highly active antiretroviral therapy in two patients with AIDS-associated Kaposi sarcoma. Am. J. Clin. Oncol..

[14] Watanabe A., Ishizuka T., Yamada M., Igawa Y., Shimizu T., Ishizuka H. (2022). Physiologically based pharmacokinetic modelling to predict the clinical effect of CYP3A inhibitors/inducers on esaxerenone pharmacokinetics in healthy subjects and subjects with hepatic impairment. Eur. J. Clin. Pharmacol..

[15] Kuepfer L., Niederalt C., Wendl T., Schlender J.F., Willmann S., Lippert J., Block M., Eissing T., Teutonico D. (2016). Applied Concepts in PBPK Modeling: How to Build a PBPK/PD Model. CPT Pharmacomet. Syst. Pharmacol..

[16] Foti R.S. (2025). Utility of physiologically based pharmacokinetic modeling in predicting and characterizing clinical drug interactions. Drug Metab. Dispos..

[17] de Zwart L., Snoeys J., De Jong J., Sukbuntherng J., Mannaert E., Monshouwer M. (2016). Ibrutinib Dosing Strategies Based on Interaction Potential of CYP3A4 Perpetrators Using Physiologically Based Pharmacokinetic Modeling. Clin. Pharmacol. Ther..

[18] Wang K., Yao X., Zhang M., Liu D., Gao Y., Sahasranaman S., Ou Y.C. (2021). Comprehensive PBPK model to predict drug interaction potential of Zanubrutinib as a victim or perpetrator. CPT Pharmacomet. Syst. Pharmacol..

[19] Zhou D., Podoll T., Xu Y., Moorthy G., Vishwanathan K., Ware J., Slatter J.G., Al-Huniti N. (2019). Evaluation of the Drug-Drug Interaction Potential of Acalabrutinib and Its Active Metabolite, ACP-5862, Using a Physiologically-Based Pharmacokinetic Modeling Approach. CPT Pharmacomet. Syst. Pharmacol..

[20] de Jong J., Sukbuntherng J., Skee D., Murphy J., O’Brien S., Byrd J.C., James D., Hellemans P., Loury D.J., Jiao J. (2015). The effect of food on the pharmacokinetics of oral ibrutinib in healthy participants and patients with chronic lymphocytic leukemia. Cancer Chemother. Pharmacol..

[21] Ou Y.C., Preston R.A., Marbury T.C., Tang Z., Novotny W., Tawashi M., Li T.K., Sahasranaman S. (2020). A phase 1, open-label, single-dose study of the pharmacokinetics of zanubrutinib in subjects with varying degrees of hepatic impairment. Leuk. Lymphoma.

[22] Chen B., Zhou D., Wei H., Yotvat M., Zhou L., Cheung J., Sarvaria N., Lai R., Sharma S., Vishwanathan K. (2022). Acalabrutinib CYP3A-mediated drug-drug interactions: Clinical evaluations and physiologically based pharmacokinetic modelling to inform dose adjustment strategy. Br. J. Clin. Pharmacol..

[23] Wagner C., Zhao P., Arya V., Mullick C., Struble K., Au S. (2017). Physiologically Based Pharmacokinetic Modeling for Predicting the Effect of Intrinsic and Extrinsic Factors on Darunavir or Lopinavir Exposure Coadministered With Ritonavir. J. Clin. Pharmacol..

[24] Arora S., Pansari A., Kilford P., Jamei M., Gardner I., Turner D.B. (2020). Biopharmaceutic In Vitro In Vivo Extrapolation (IVIVE) Informed Physiologically-Based Pharmacokinetic Model of Ritonavir Norvir Tablet Absorption in Humans Under Fasted and Fed State Conditions. Mol. Pharm..

[25] Colbers A., Greupink R., Litjens C., Burger D., Russel F.G. (2016). Physiologically Based Modelling of Darunavir/Ritonavir Pharmacokinetics During Pregnancy. Clin. Pharmacokinet..

[26] Marzolini C., Rajoli R., Battegay M., Elzi L., Back D., Siccardi M. (2017). Physiologically Based Pharmacokinetic Modeling to Predict Drug-Drug Interactions with Efavirenz Involving Simultaneous Inducing and Inhibitory Effects on Cytochromes. Clin. Pharmacokinet..

[27] Ke A., Barter Z., Rowland-Yeo K., Almond L. (2016). Towards a Best Practice Approach in PBPK Modeling: Case Example of Developing a Unified Efavirenz Model Accounting for Induction of CYPs 3A4 and 2B6. CPT Pharmacomet. Syst. Pharmacol..

[28] Litou C., Turner D.B., Holmstock N., Ceulemans J., Box K.J., Kostewicz E., Kuentz M., Holm R., Dressman J. (2020). Combining biorelevant in vitro and in silico tools to investigate the in vivo performance of the amorphous solid dispersion formulation of etravirine in the fed state. Eur. J. Pharm. Sci..

[29] Stader F., Courlet P., Kinvig H., Battegay M., Decosterd L.A., Penny M.A., Siccardi M., Marzolini C. (2021). Effect of ageing on antiretroviral drug pharmacokinetics using clinical data combined with modelling and simulation. Br. J. Clin. Pharmacol..

[30] Hsu A., Granneman G.R., Witt G., Locke C., Denissen J., Molla A., Valdes J., Smith J., Erdman K., Lyons N. (1997). Multiple-dose pharmacokinetics of ritonavir in human immunodeficiency virus-infected subjects. Antimicrob. Agents Chemother..

[31] Sekar V., Lavreys L., Van de Casteele T., Berckmans C., Spinosa-Guzman S., Vangeneugden T., De Pauw M., Hoetelmans R. (2010). Pharmacokinetics of darunavir/ritonavir and rifabutin coadministered in HIV-negative healthy volunteers. Antimicrob. Agents Chemother..

[32] Wang J., Zhang Z.Y., Lu S., Powers D., Kansra V., Wang X. (2019). Effects of rolapitant administered orally on the pharmacokinetics of dextromethorphan (CYP2D6), tolbutamide (CYP2C9), omeprazole (CYP2C19), efavirenz (CYP2B6), and repaglinide (CYP2C8) in healthy subjects. Support. Care Cancer.

[33] Boffito M., Jackson A., Lamorde M., Back D., Watson V., Taylor J., Waters L., Asboe D., Gazzard B., Pozniak A. (2009). Pharmacokinetics and safety of etravirine administered once or twice daily after 2 weeks treatment with efavirenz in healthy volunteers. J. Acquir. Immune Defic. Syndr..

[34] Cox D.S., Rehman M., Khan T., Ginman K., Salageanu J., LaBadie R.R., Wan K., Damle B. (2023). Effects of nirmatrelvir/ritonavir on midazolam and dabigatran pharmacokinetics in healthy participants. Br. J. Clin. Pharmacol..

[35] Abel S., Jenkins T.M., Whitlock L.A., Ridgway C.E., Muirhead G.J. (2008). Effects of CYP3A4 inducers with and without CYP3A4 inhibitors on the pharmacokinetics of maraviroc in healthy volunteers. Br. J. Clin. Pharmacol..

[36] Kakuda T.N., Abel S., Davis J., Hamlin J., Schöller-Gyüre M., Mack R., Ndongo N., Petit W., Ridgway C., Sekar V. (2011). Pharmacokinetic interactions of maraviroc with darunavir-ritonavir, etravirine, and etravirine-darunavir-ritonavir in healthy volunteers: Results of two drug interaction trials. Antimicrob. Agents Chemother..

[37] Rowland Yeo K., Gil Berglund E., Chen Y. (2024). Dose Optimization Informed by PBPK Modeling: State-of-the Art and Future. Clin. Pharmacol. Ther..

[38] Li J., Zhao M., He P., Hidalgo M., Baker S.D. (2007). Differential metabolism of gefitinib and erlotinib by human cytochrome P450 enzymes. Clin. Cancer Res..

[39] Moltó J., Rajoli R., Back D., Valle M., Miranda C., Owen A., Clotet B., Siccardi M. (2017). Use of a physiologically based pharmacokinetic model to simulate drug-drug interactions between antineoplastic and antiretroviral drugs. J. Antimicrob. Chemother..

[40] (2022). Imbruvica (Ibrutinib).

[41] (2022). Calquence (Acalabrutinib).

[42] (2023). Brukinsa (Zanubrutinib).

[43] Efavirenz (Sustiva). https://www.ema.europa.eu/en/documents/product-information/sustiva-epar-product-information_en.pdf.

[44] Etravirine (Intelence). https://www.ema.europa.eu/en/documents/product-information/intelence-epar-product-information_en.pdf.

[45] Yanakis L.J., Bumpus N.N. (2012). Biotransformation of the antiretroviral drug etravirine: Metabolite identification, reaction phenotyping, and characterization of autoinduction of cytochrome P450-dependent metabolism. Drug Metab. Dispos..

[46] Zhang H., Ou Y.C., Su D., Wang F., Wang L., Sahasranaman S., Tang Z. (2021). In vitro investigations into the roles of CYP450 enzymes and drug transporters in the drug interactions of zanubrutinib, a covalent Bruton’s tyrosine kinase inhibitor. Pharmacol. Res. Perspect..

[47] Podoll T., Pearson P.G., Kaptein A., Evarts J., de Bruin G., Emmelot-van Hoek M., de Jong A., van Lith B., Sun H., Byard S. (2023). Identification and Characterization of ACP-5862, the Major Circulating Active Metabolite of Acalabrutinib: Both Are Potent and Selective Covalent Bruton Tyrosine Kinase Inhibitors. J. Pharmacol. Exp. Ther..

[48] Gupta A., Zhang Y., Unadkat J.D., Mao Q. (2004). HIV protease inhibitors are inhibitors but not substrates of the human breast cancer resistance protein (BCRP/ABCG2). J. Pharmacol. Exp. Ther..

[49] Profit L., Eagling V.A., Back J.D. (1999). Modulation of P-glycoprotein function in human lymphocytes and Caco-2 cell monolayers by HIV-1 protease inhibitors. Aids.

[50] Gao N., Zhang X., Hu X., Kong Q., Cai J., Hu G., Qian J. (2022). The Influence of CYP3A4 Genetic Polymorphism and Proton Pump Inhibitors on Osimertinib Metabolism. Front. Pharmacol..

