# Supplementary material for: Physiologically Based Pharmacokinetic Modeling to Assess Antiretroviral–BTK Inhibitor Interactions and Provide Recommendations for Co-Administration Regimens
Source: Pharmaceutics. 2026 Apr 10;18(4):465. doi: 10.3390/pharmaceutics18040465 (PMC13118766; doi:10.3390/pharmaceutics18040465)
Supplement: Supplementary file 1 [file pharmaceutics-18-00465-s001.zip › pharmaceutics-4206797-supplementary.pdf]

pharmaceutics

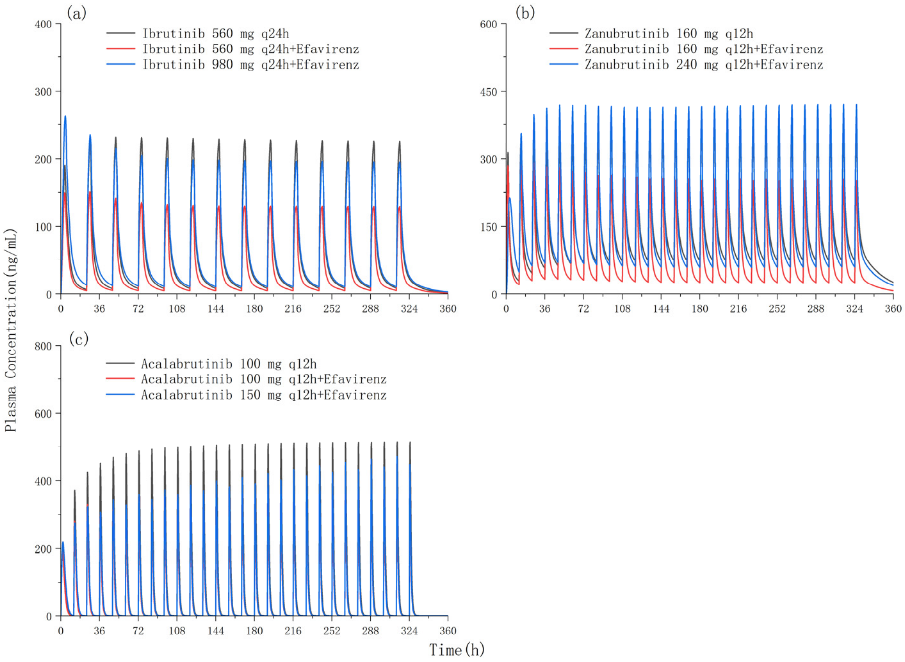


**Figure S1.** Simulated plasma concentration–time profiles of multiple doses (14 days doses) of (a) ibrutinib, (b) zanubrutinib, and (c) acalabrutinib in the absence and presence of efavirenz (600 mg once daily).

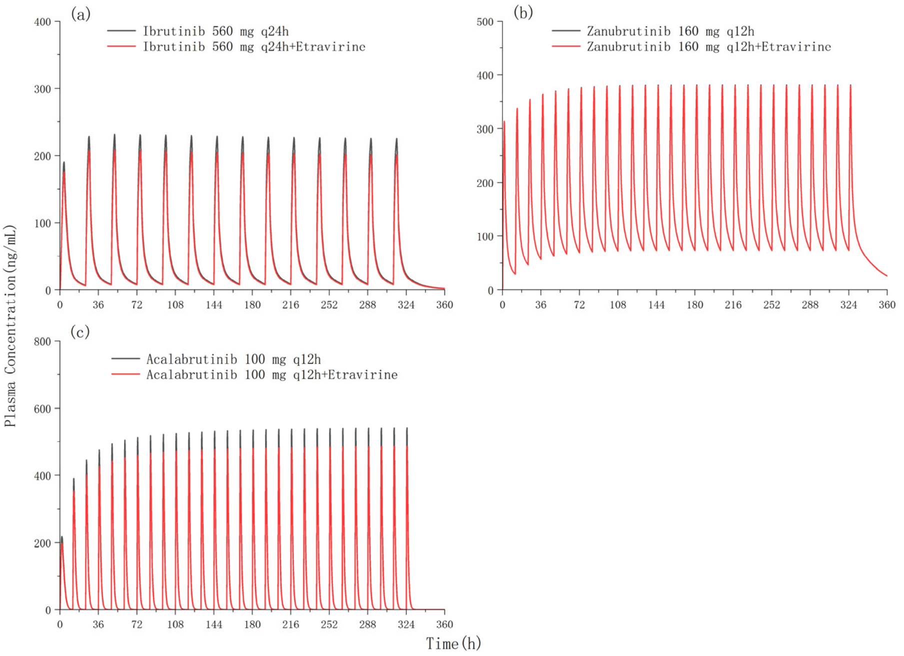


**Figure S2.** Simulated plasma concentration–time profiles of multiple doses (14 days doses) of (a) ibrutinib, (b) zanubrutinib, and (c) acalabrutinib in the absence and presence of etravirine (200 mg twice daily).

 https://doi.org/10.3390/pharmaceutics18040465
